# Factors Affecting Job Motivation among Health Workers: A Study From Iran

**DOI:** 10.5539/gjhs.v7n3p153

**Published:** 2014-11-25

**Authors:** Abbas Daneshkohan, Ehsan Zarei, Tahere Mansouri, Khadije Maajani, Mehri Siyahat Ghasemi, Mohsen Rezaeian

**Affiliations:** 1 Department of Public Health, School of Health, Shahid Beheshti University of Medical Sciences, Tehran, Iran; 2 Departmet of Health Education and Health Promotion, School of Health, Iran University of Medical Sciences, Tehran, Iran; 3 Department of Social Medicine, Occupational Environmental Research Center, School of Medicine, Rafsanjan University of Medical Sciences, Rafsanjan, Iran

**Keywords:** job motivation, health workers, Iran

## Abstract

**Objective:**

Human resources are the most vital resource of any organizations which determine how other resources are used to accomplish organizational goals. This research aimed to identity factors affecting health workers’ motivation in Shahid Beheshti University of Medical Sciences (SBUMS).

**Method:**

This is a cross-sectional survey conducted with participation of 212 health workers of Tehran health centers in November and December 2011. The data collection tool was a researcher-developed questionnaire that included 17 motivating factors and 6 demotivating factors and 8 questions to assess the current status of some factors. Validity and reliability of the tool were confirmed. Data were analyzed with descriptive and analytical statistical tests.

**Results:**

The main motivating factors for health workers were good management, supervisors and managers’ support and good working relationship with colleagues. On the other hand, unfair treatment, poor management and lack of appreciation were the main demotivating factors. Furthermore, 47.2% of health workers believed that existing schemes for supervision were unhelpful in improving their performance.

**Conclusion:**

Strengthening management capacities in health services can increase job motivation and improve health workers’ performance. The findings suggests that special attention should be paid to some aspects such as management competencies, social support in the workplace, treating employees fairly and performance management practices, especially supervision and performance appraisal.

## 1. Introduction

Human resources determine the use of other available resources. They are the most important category of the health systems inputs (World Health Organization [WHO], 2000). Despite of the importance, human resource management systems are facing serious problems in developing countries (Adano, 2006). Evidence suggests that the inheritance of constant under-investment in human resources could lead to the underperformance of health systems (Razee, Whittaker, Jayasuriya, Yaple, & Brentnall, 2012). Therefore, health worker retention is crucial for health system performance (Willis-Shattuck et al., 2008).

It is stated that health worker motivation is the main determinant of health worker retention and health sector performance (Peters, Chakraborty, Mahapatra & Steinhardt, 2010). Job Motivation is defined as “the willingness to exert and maintain an effort towards organizational goals” (Mathauer & Imhoff, 2006) and motivated health workforce are more likely to apply their knowledge to the real delivery of health care (Prytherch et al., 2013). Given the current challenges such as poor working conditions, personal safety concerns and inadequate equipments, job motivation could play a key role in productivity of human resources for health in developing countries (Luoma, 2006; Mutale, Ayles, Bond, Mwanamwenge & Balabanova, 2013; WHO, 2006a; Mbilinyi, Daniel & Lie, 2011). The key issue in this regard is to identify factors that affecting employees’ motivation.

The WHO stated that the Islamic Republic of Iran has achieved the substantial progress in providing access to primary health care for all the people during recent years. The health indicators of Iran now demonstrate a steady improvement getting near to those of developed countries. However, poor human resource management is one of the most important health system challenges of the country (WHO, 2011a; WHO, 2006b; WHO, 2009). This research aimed to identity factors affecting health workers’ motivation in SBUMS.

## 2. Methods

### 2.1 Study Design

This study was a Cross-Sectional one conducted in November and December 2011. The study population were health workers of health centers covered by SBUMS which is responsible for health care provision for one third of all population of Tehran, Capital of Iran. The university covers 55 health centers in Tehran. Due to financial and time constraints 20 health centers were selected using random sampling. All health workers of selected centers were participated in the study. 250 questionnaire were distributed and 240 (96%) were returned, 28 dropped from further analysis because of missing responses over 20%.

### 2.2 Measurement Instrument

After doing a literature review and studying WHO documents, especially the World Health Report 2006 in which health workforce management strategies have been introduced (WHO, 2006a), a self-administered questionnaire consisting of three parts was developed. The first part included demographic characteristics and the second part included 17 motivating and 6 demotivating factors according to the Likert scale with a range of five points from 1 to 5. The final section of the questionnaire consisted of 8 questions for determining status of some variables consisting of job description, rewards and incentives, selection criteria for on-the-job training, supervision, performance appraisal, appropriateness of training courses in responding to job needs, training for management tasks and appreciation in the workplace.

### 2.3 Data Analysis

The questionnaire’s validity was examined using the views of 15 scholars and experts in management discipline and their constructive views were considered in modifying the tool. The reliability coefficient of the questionnaire was calculated by Cronbach’s alpha and estimated to be 0.87. The results of Kolmogorov-Smirnov test showed that data was not distributed normally in 0.05 level and therefore, we used non-parametric statistics for analyzing relation between motivational factors and demographic variables in SPSS software. In all statistical analyses the P-value smaller than 0.05 was considered significant.

## 3. Results

### 3.1 Demographic Characteristics of Study Sample

Eighty one percent of study population (n=172) were female and 19% (n=40) were male. The mean age of them was 36.8±8.8. Age group 40 and more (43%) had the most frequency. Furthermore, Fifty two percent of respondents (n=111) had bachelor’s degree and 75% of them (n=159) were married. In 41% (n=87) of study population years of practice was between 10-19 years (Table 1).

**Table 1:** Demographic characteristics of the study population

Variable	No	%
Sex:		
Male	172	81.1
Female	40	18.9

Age group:		
<30	65	31.7
30–39	51	24.9
≥40	89	43.4

Marital status:		
Married	159	75
Single and divorced	53	25

Education:		
Diploma and under diploma	27	12.7
Associate degree	36	17
Bachelor degree	111	52.4
Master and Doctorate	38	17.9

Years of practice		
<10	77	36.3
10–19	87	41
≥ 20	48	22.7

### 3.2 Motivating and Demotivating Factors

Mean scores of motivating and demotivating factors are given in rank order in tables 2 and 3. The results show that good management (4.58 ± 0.68) was the most important motivator for health workers followed by support from supervisors and managers when occurring problems, good working relations with colleagues and fair treatment from supervisors and managers, whereas resource availability, financial incentives, continuing education and non-financial welfare benefits had the least effects on motivating the study population.

**Table 2:** Mean scores and standard deviations of factors motivating health workers

Factor	Average Score	SD
good management	4.58	0.68
Support from supervisors and managers when occurring problems	4.47	0.83
Good working relations with colleagues	4.44	0.69
Fair treatment from supervisors and managers	4.41	0.85
Job security	4.40	0.87
Good working relations with supervisors	4.37	0.72
Career development (in regards to the possibility to specialize or be promoted)	4.31	0.96
Recognition and appreciation	4.31	0.85
Clarity of rules and guidelines	4.26	0.87
Social status of job	4.21	0.79
Nature of work	4.20	0.79
Good physical conditions	4.14	0.88
Participation in decision making	4.14	0.78
Resource availability	4.09	0.82
Financial rewards (salary and overtime)	4.02	1.05
Continuing education	4.01	0.90
Non-financial welfare benefits	3.91	1.14

**Table 3:** Mean scores and standard deviations of factors demotivating health workers

Factor	Average score	SD
Unfair treatment	4.56	0.67
Poor management	4.35	0.87
Lack of recognition and appreciation	4.18	0.85
Subjective performance appraisal	4.12	0.85
Lack of job description	3.95	0.85
Difficult living conditions	3.61	0.99

Unfair treatment was the most powerful demotivating factor (4.56 ± 0.67). Other demotivators were poor management, lack of recognition and appreciation, subjective performance appraisal, lack of job description and difficult living conditions.

### 3.3 Current Status of Some Factors

In response to the third section of questionnaire 54% of employees stated that they didn’t have job descriptions. Moreover, 44.2% of those who had job descriptions considered them as being not update and inappropriate with new working conditions. In the opinion of 83.7% of them rewards and incentives were distributed unfairly. The results highlighted that 49.7% of health workers believed that existing schemes for supervision were unhelpful in improving their performance. Similarly, 49.7% of them maintained that the methods of performance appraisal were subjective and 40% of health workers thought that training courses were inappropriate. Furthermore 83% of health workers believed that encouragement and appreciation practices were not effective and adequate. Mann-Whitney Test results showed that financial incentives was significantly more motivating for singles and divorced compared to the married employees(p=0.036).

## 4. Discussion

The results of the current study highlighted that the most important motivating factor for health workers was good management. This shows that health workers have positive attitudes towards the importance of management practices in the organization. In a systematic review of motivation and retention of health workers in developing countries, the important role of management as a motivational factor was highlighted. The reviewed studies consistently provided views from health workers who declared that leadership and management skills of their supervisors were insufficient and this led to de-motivation of the personnel (Willis-Shattuck et al., 2008).Similarly, participants in a study of conflict among Iranian hospital nurses believed that some managers’ behavior influenced the increase of conflict occurrence. Some managers were seen to have mistreated staff, suddenly changed their styles and failed to understand and support their staff (Dehghan Nayeri & Negarandeh, 2009). Moreover, Dehghan Nayeri, Nazari, Salsali and Ahmadi (2005) in their study concluded that managers’ role was an important factor affecting nurses’ productivity.

Evidence suggests that since organizational needs always go beyond available resources therefore, no organization can do well without high-quality management (Management Sciences for Health [MSH], 2010a). High-quality management can work as a glue which holds firmly all the internal parts of an organization together, leads to a constructive work situation, and supports premium services (MSH, 2010b). The WHO has stated that weaknesses in managerial capacity always act as a limitation to accomplish the Millennium Development Goals (MDGs) (WHO, 2007). It seems that all around the world the lack of management skills performs as the single most important obstacle to improve health outcomes (Peterson, et al., 2011). When an organization manages its workforce sensibly, the result is satisfied and motivated personnel who deliver high quality health care (MSH, 2009).

The second and the third motivating factors for health workers in this study were supervisors’ and managers’ support and good working relations with colleagues, respectively. Evidence suggests that supportive manager is a vital aspect for retention (WHO, 2006c). Health personnel are more motivated when their managers provide them with a clear sense of vision and mission, listen to them and make them feel recognized and valued no matter what their job (WHO, 2006a). A study conducted by Stilwell (2001) in Zimbabwe concluded that health staff in remote areas by having good leadership and supportive management were motivated well in spite of hard working conditions and low financial incentives.

The fact that the financial rewards was ranked 15th out of 17 motivation factors by respondents, match with Hezberg’s two-factor theory in which salary is considered as a hygiene factor and not a motivating factor (Gibson, Ivancevich, Donnelly, & Konopaske, 2012). Similarly, health workers of two Indian states ranked good income as the third least important characteristic of an ideal job (Peters et al., 2010). However, Pakistani physicians and Malian health workers ranked salary and good pay as the first and the second important motivating factor, respectively (A. Malik, Yamamoto, Souares, Z. Malik, & Sauerborn, 2010; Dieleman, Toonen, Toure, & Martineau, 2006). Generally, money is rarely the most important motivator. The results of a study conducted in Pakistan highlighted that although financial incentives are important but not sufficient enough to improve health workers’ performance (Malik et al., 2010). Moreover, although health workers need to receive a living wage (WHO, 2006a), evidence suggests that too much focus on financial incentives to motivate individuals in the public sector might have some negative impacts (Malik et al., 2010).

The research results revealed that unfair treatment was the first demotivating factor in our study. Similarly, health workers in Tanzania indicated that perceived unfairness in areas such as promotions, salary levels and access to training and upgrading had negative impact on their motivation to work. Nevertheless, in Vietnam low salaries was the main discouraging factor (Songstad, Rekdal, Massay, & Blystad, 2011).

Generally, one of the most important concerns of employees is that are they being treated fairly? Their perception of fairness regarding areas such as salary and total compensation, daily application of personnel policies and recognition of contributions has a powerful impact on their performance and retention (MSH, 2010a; Dieleman, Vietcuong, Anh, & Martineau, 2003). Evidence shows that a sense of unfairness can decrease motivation to work (WHO, 2006a). Some sources of such sense are lack of sound promotion systems, lack of systematic organizational rewards for hard work and incapability to choose between individuals on merit (WHO, 1993).

In our study unfairness of rewards and incentives in view of vast majority of study population and subjectivity of performance appraisal in view of nearly half of them are signs of perceived unfairness. Malian health workers also believed that managers’ decisions in training and performance appraisal has not been transparent (Dieleman et al., 2006). Research findings indicate that perceived unfairness at work has negative effects on employees’ health and well being. In this regard an association between low justice and cardiovascular diseases has been reported (Shibaoka et al., 2010). Furthermore, review of evidence suggests that low perceived justice has been associated with stress, inflammation, sleeping problems, cognitive impairments and work absenteeism (Elovainio, Heponiemi, Sinervo, & Magnavita, 2010).

The finding that average score of unfair treatment as a demotivator is higher than that of fair treatment as a motivating factor is consistent with the fact that the negative impacts of unfairness are significantly stronger than the positive impacts of fairness (Chan, 2011). The matter emphasizes on necessity of high attention of managers to indicators of perceived unfairness in different ways, including regular meetings and periodical employee attitude surveys.

Poor management was the second demotivator of the respondents. In Mali, health workers cited poor management as a demotivating factor (Dieleman et al., 2006). Kebriaei and Moteghedi (2009) in their study reported that satisfaction of community health workers in Zahedan district (south-east of Iran) with management was also low (Kebriaei & Moteghedi, 2009). In fact, many health workers are demotivated due to poor human resource management practices (Mathauer & Imhoff, 2006).

Lack of recognition and appreciation and subjective performance appraisal were other discouraging factors in our study. One third of Iranian nurses participated in a survey of performance appraisal outcomes and it’s relation with motivation believed that the outcomes were negative (Taghavi Larijani, Parsa Yakta, Kazemnejad, & Mazaheri, 2006). According to majority of research participants in a study conducted by Najafi, Hamidi, Ghiasi, Shahhoseini and Emami (2011) performance appraisal acts as an important factor in job motivation, but 67.42% of participants declared that they have never benefited from performance appraisal. According to 45.90% of them, it is because of the discrimination, injustice and favoritism (Najafi et al., 2011). This matter also had a negative effect on motivation of hospital personnel in Kenya (Mbindyo, Gilson, Blaauw, & English, 2009). An astonishing relation between recognition and motivation among staff is also reported by MSH (MSH, 2010a). The two management functions are directly related to performance management system, including performance appraisal. It is stated that unclear or unfair performance appraisal systems are among important factors contributing to the sense of injustice and job stress (Mullins, 2005; WHO, 2003). Whereas the function (performance appraisal), when conducted equitably, can improve on employees’ morale, motivation and self-esteem (WHO, 2011b).

Lack of job description and difficult living conditions were two final demotivators in our study. Study of barriers to motivation in Iranian nurses identified lack of clear job description as a barrier to job motivation (Oshvandi et al., 2008). It is shown that clear job descriptions are consistently associated with enhanced attainment of work goals (WHO, 2006a).

Unhelpfulness of supervision schemes in performance improvement in view of nearly half of respondents is another issue that should be paid more attention. Similarly, in Benin and Kenya health workers believed that existing supervision practices were ineffective (Mathauer & Imhoff, 2006). Also in Zambia, health workers were facing unsupportive management and supervision (Mutale et al., 2013). According to WHO report supportive supervision alongside with clear job descriptions and feedback on performance is one of the most effective instruments to improve the competence of individual health workers (WHO, 2006a).

Financial incentives as a motivational factor was ranked higher by singles and divorced than by married employees perhaps the reason for this is that the singles prepare for marriage and the divorced should cover the cost of divorce whilst the married employees usually have greater degree of financial stability.

Our study has limitations. This research used quantitative method for data gathering. Qualitative data could lead to a better understanding of motivating and demotivating factors. The study also did not measure motivation and performance of health workers so it is not clear how the studied factors affect on their actual performance in the field.

## 5. Conclusion

Given the results of present study strengthening managerial capacity in health services should be emphasized in order to increase motivation of health workers. Considering the negative impact of perceived unfairness on motivation, managers must apply personnel policies fairly on day to day basis, communicate appropriate information about equity to all employees and act in a transparent manner. Also, more attention needs to be given to developing a fair and objective performance appraisal system and implementing supportive supervision strategies.
